# Case Report: ‘Z’ osteotomy - a novel technique of treatment in Blount’s disease

**DOI:** 10.12688/f1000research.6770.1

**Published:** 2015-11-12

**Authors:** Raju Karuppal, Rahul Mohan, Anwar Marthya, Gopakumar TS, Sandhya S

**Affiliations:** 1 Department of Orthopedics, Govt. Medical College Kozhikode, Kerala Health University, Kerala, 673008, India; 2 IQRAA International hospital and research centre, Kozhikode, Kerala, 673008, India; 3 Department of Ophthalmology, Govt. Medical College Kozhikode, Kerala Health University, Kerala, 673008, India

**Keywords:** Infantile tibia vara, Blount’s disease, Z tibial osteotomy

## Abstract

Blount’s disease is a progressive form of genu varum due to asymmetrical inhibition of the postero medial portion of the proximal tibial epiphysis. The surgical treatments involved in correction of Blount’s disease are often technically demanding, complicated procedures.  These procedures can lead to prolonged recovery times and poor patient compliance. In such a context we are suggesting “fibulectomy with Z osteotomy” of the proximal tibia, a relatively simple and highly effective technique. This technique is based on correcting the mechanical axis of the lower limb thereby restoring growth from the medial physis of proximal tibia. We have used a new surgical technique, which includes fibulectomy followed by a Z-shaped osteotomy. We have used this simple technique in a 5 year-old boy with unilateral Blount’s disease. The femoro-tibial angle was corrected from 18.2° of varus to 4.2° of valgus. The angular correction obtained after operation was 22°. There were no postoperative complications. This technique has the advantages of correcting both angular and rotational deformities simultaneously.  The purpose of this case study is to introduce a new surgical technique in the treatment of Blount’s disease.

## Case report

**Figure 1.**
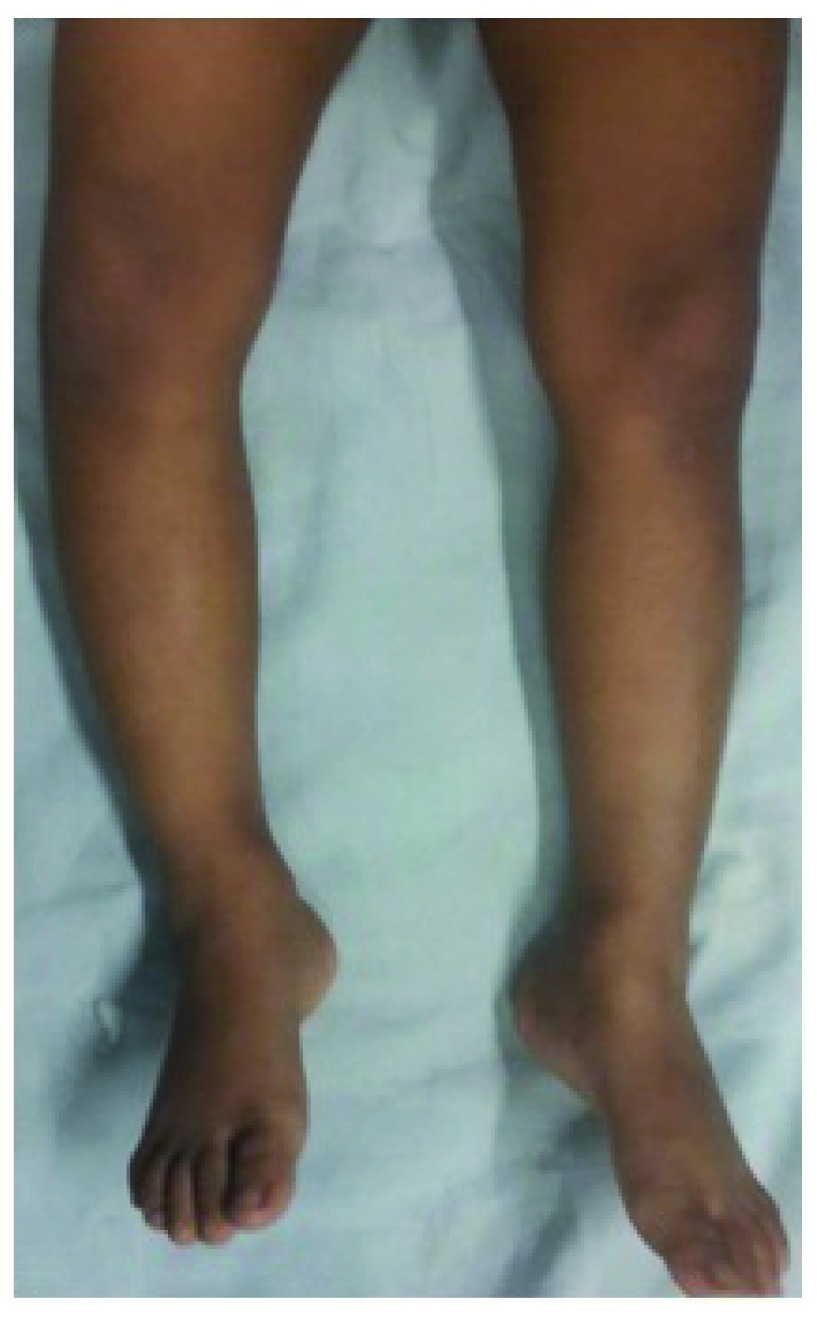
Clinical picture shows right sided tibia vara.

**Figure 2.**
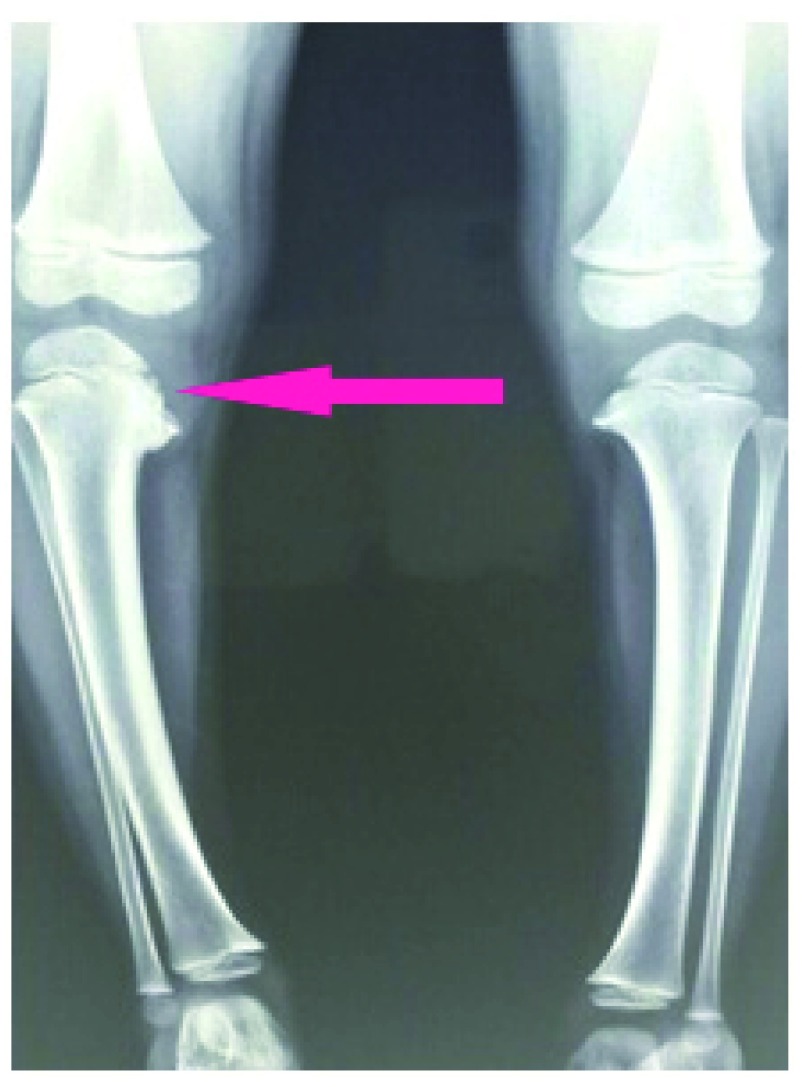
X-ray shows depression of medial tibial plateau with beaking of posteromedial tibial metaphysis.

**Figure 3.**
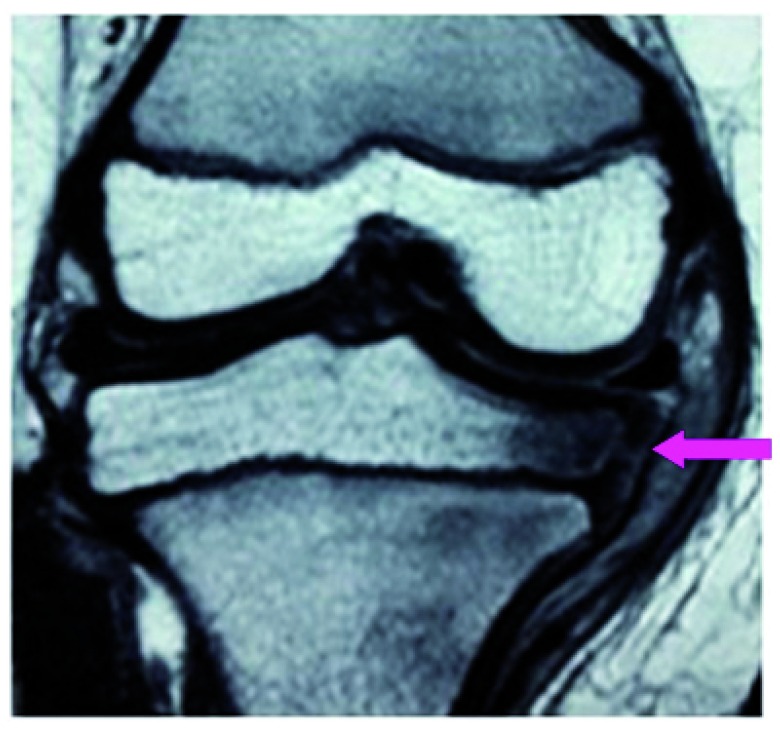
MRI (coronal PD fat sat image) shows wedge shaped medial epiphysis and deformed physis with altered signal intensity.

A 5 year-old Indian male child presented in June 2013 with unilateral right sided genu varum ( Figure 1) noted since January 2013. The deformity was gradually progressing in character. There was no significant history of trauma or infection. Clinical examination showed 18.2° of varus and 10° of intortion of tibia. Biochemical investigations were normal. X-ray showed depression of medial tibial plateau with beaking of posteromedial tibial metaphysis ( Figure 2) MRI showed an irregular medial physeal line, postero-medial depression, thinning of medial epiphyseal cartilage and concomitant increase in the joint space ( Figure 3). As the child is already 5 years old, to achieve a rapid complete correction surgical treatment was opted. Of the many surgical options like wedge osteotomy and ilizarov correction have its own many demerits. Hence we decided for Z osteotomy which will correct the angular and rotational deformities. It also has predictable result and potential for minimal complications.

## Operative technique of Z osteotomy

Pre-operative planning includes quantifying the tibio-femoral angle from standing antero-posterior X-ray of both lower limbs. The surgical aim is to achieve a correction of 5° to 7° of valgus. The rotational correction needed should also be assessed clinically. The patient should be in supine position. A tourniquet is applied and a fibular osteotomy is performed at the middle of the fibula. The proximal shaft of the tibia is then exposed through a 5 cm long incision just below the tibial tuberosity in the midline.

For the left tibial varus deformity, the upper horizontal limb of the Z osteotomy starts at the medial border of tibia, one finger breadth below the tibial tuberosity, to the anterior border of the tibia. The vertical limb descends down from this point along the anterior border for a same distance of the upper horizontal limb. The lower horizontal limb of the Z osteotomy starts from this point horizontally to the lateral border of the tibia. (For the right tibial deformity, the reverse Z osteotomy is made).

**Figure 4.**
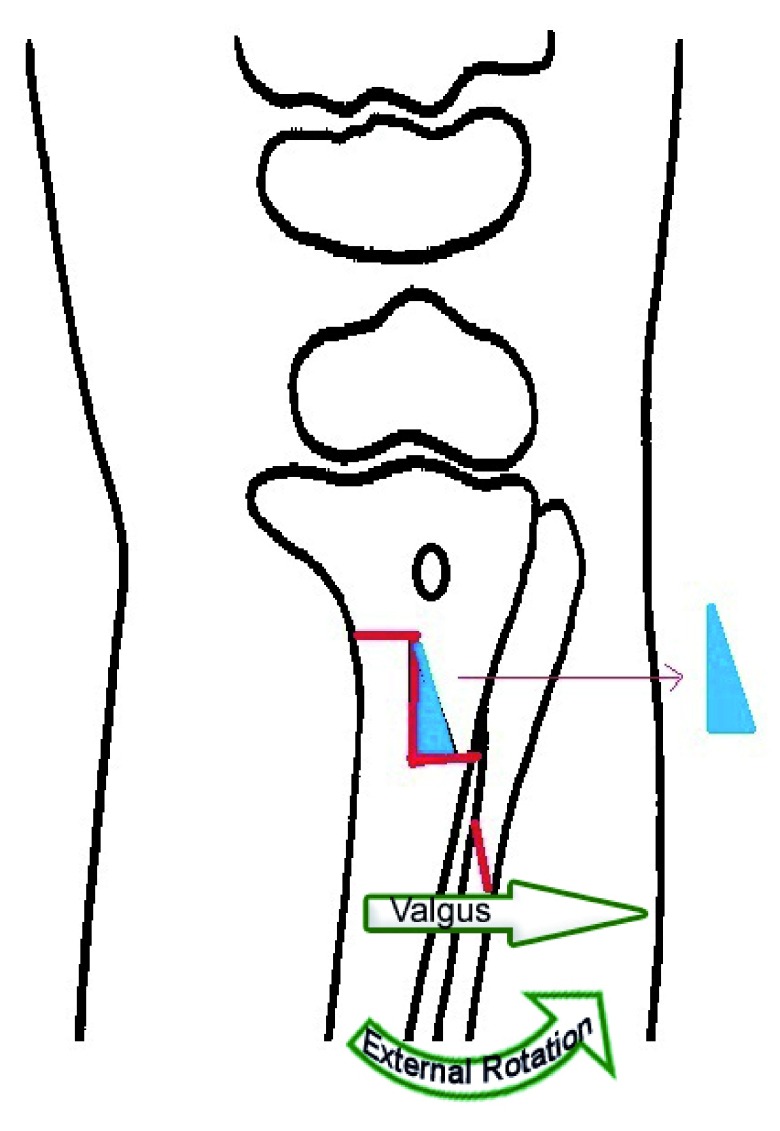
Technique of Z-osteotomy - Z shape is marked on the proximal tibia by drill holes which are connected by an osteotome to complete the osteotomy (Shown in red line). Wedge of bone is removed (Shown in blue shape) and the distal tibia is derotated and then re-engaged in the corrected position. The red line shown on the fibula is the fibular osteotomy.

**Figure 5.**
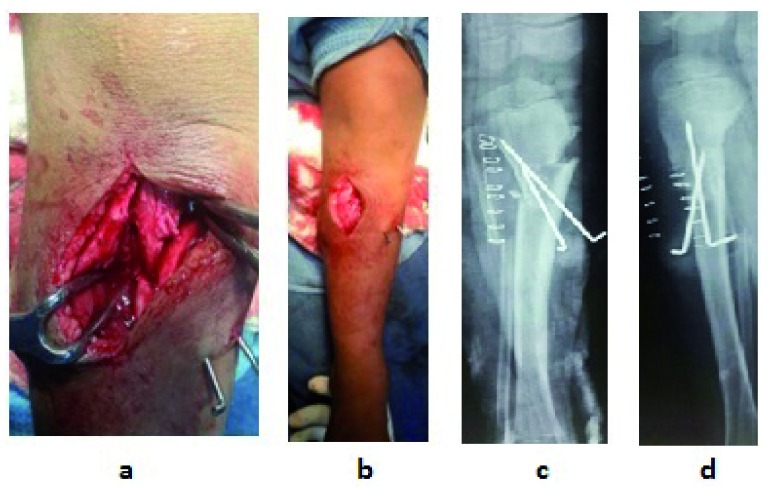
In-operative picture shows the osteotomy site and post-operative X-rays. **a**. Close-up view of correction of deformity by Z osteotomy and stabilization with 2 K wires, **b**. Varus deformity is corrected to normal alignment, **c**. Immediate post-operative X-ray anterior posterior view, **d**. Immediate post-operative X-ray lateral view.

A Z shape is marked on the proximal tibia by drill holes which are connected by an osteotome to complete the osteotomy. The width of the wedge to be removed is calculated as 1mm for each 1° of angle to be corrected. A wedge of bone is removed between the vertical and lateral horizontal limb of the Z osteotomy ( Figure 4) and the distal tibia is derotated and angulated laterally then re-engaged in the corrected position. The osteotomy site is then stabilized with one or two k wires ( Figure 5). The final alignment is confirmed by an X-ray image intensifier. The leg is then immobilized in a long-leg cast. In the presented case, this was worn for eight weeks, after which weight-bearing was allowed. The postoperative period was uneventful.

**Figure 6.**
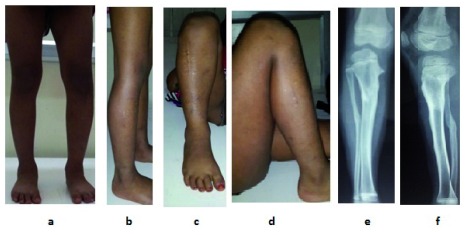
Clinical picture of correction at 2 years follow-up. **a**. Standing front view shows deformity is corrected to normal and comparable to the opposite side, **b**. Standing side view shows deformity is corrected, **c**. Front view of leg in knee flexed position, **d**. Side view of leg in knee flexed position, **e** & **f**. X-ray AP and lateral view shows consolidation of osteotomy site with normal tibial mechanical axis.

The femoro-tibial angle was corrected from 18.2° of varus to 4.2° of valgus. The angular correction obtained after operation was 22°. There were no major complications or any neurological problems in our case. The result was favorably comparable with other reported surgical techniques. At the last follow-up in May 2015 child had maintained the correction of angular and rotational alignment ( Figure 6).

## Discussion

Infantile tibia vara or Blount’s disease was the first described by Erlacher in 1922 ^1^. The three-dimensional complex deformity of Blount’s disease includes varus, internal rotation, and (sometimes) procurvatum ^2^. The progressive varus deformity in Blount’s disease is thought to be due to repetitive, compressive injury of the proximal tibial growth plate medially with relative overgrowth of the lateral tibial physis ^3^. The varus deformity may improve by the age of 4 years; hence operation should be delayed unless significant lateral thrust or other symptoms develop. The spontaneous resolution of the varus deformity in Blount’s disease is rare ^4^.

A rapid complete correction would generally be achieved by an osteotomy. It is not advisable to operate on physis to avoid growth disturbances. The commonly used osteotomies are closing and opening wedges at proximal tibia. Other alternatives are Dome and chevron-type osteotomies ^5^. The results of closing-wedge, proximal tibial osteotomy was published by Laurencin *et al.* ^6^. It has many disadvantages like fracture of the medial cortex, which would produce over correction and shortening of the limb. Martin *et al.* ^7^ described the result of Opening-wedge-type osteotomy. The disadvantages of this technique include undercorrection of the internal tibial torsion and instability at the osteotomy site, which requires rigid internal fixation. The disadvantages of external fixation for stabilising osteotomies for tibia vara are longer associated consolidation times, unsightly scars and the need for expensive, complex devices ^8^. It also has other demerits, like pin-track infections and postoperative neurapraxia.

The best way to obtain correction should be a simple procedure as near to the deformity (as high in the tibia as possible) to promote rapid union and quick remodeling ^9^.

The Z osteotomy of tibia as it has many advantages. Biomechanically, it is more stable than the closing and opening wedge osteotomies because of the special geometry of the osteotomy. Rotational deformity can be simultaneously corrected without affecting the stability and contour of the bone. Bone healing is predicted to be better because of the larger surface area at the osteotomy site. In the surgeon’s perspective it is simple to learn and perform. Only one or two K wires for short term fixation are required because of the inherent stability of the Z shape of the osteotomy, hence a second surgery for the implant removal can be avoided as well. The correction achieved following Z osteotomy is based on the principle of correcting mechanical axis of lower limb thereby restoring growth from the medial tibial physis. As such this procedure does not have any contraindications or limitations similar to other corrective osteotomies.

## Conclusion

The Z osteotomy of tibia is a useful simple technique for the correction of tibia vara in Blount’s disease, which has not been previously described in the literature. The Z osteotomy achieves angular and rotational correction of the deformity requiring minimal internal fixation. The amount of correction can be predetermined by appropriate wedge dimensions. It is an easy technique to learn and perform. It allows correction of the deformity while maintaining length, restoring joint alignment and mechanical axis of the limb.

## Consent

We have obtained informed consent for publication of clinical details and images from the parent of the child.
