# Supplementary material for: Case Report: ‘Z’ osteotomy - a novel technique of treatment in Blount’s disease
Source: F1000Res. 2015 Nov 12;4:1250. [Version 1] doi: 10.12688/f1000research.6770.1 (PMC4972087; doi:10.12688/f1000research.6770.1)
Supplement: Peer review history

# Peer review: Referee response for version 1

**DOI:** 10.5256/f1000research.7274.r14967

**Published:** 2016-08-01

**Authors:** Sandeep Vijayan, Hitesh Shah

**Affiliations:** 1 Kasturba Medical College , Manipal, Karnataka, India

**Positive points:**

 The article is well designed and authored mentioning the surgical management of varus deformity in Blount’s disase. The case report and surgical steps are clearly described. The described method appears to be a simple and the clinical and radiological correction achieved by the described procedure looks good and hence could contribute as an additional technique in the management of Blount’s disease.

**Some of my queries and suggestions are as follows:**

1. Case report (line 5) – “Clinical examination showed 18.2 deg of varus”. It would be better if authors can round off the deformity to 18 deg, as assessing 0.2 deg of varus CLINICALLY would be really challenging.
2. Case report (line 10) – Even though, X ray shows medial joint space widening, the MRI does not show concomitant increase in the joint space. Hence that sentence may be modified.
3. While de-rotating and taking the distal fragment into valgus, the superomedial corner of the distal fragment is seen to be unduly prominent (on radiograph). Considering the fact that the medial border of tibia is subcutaneous, whether this undue prominence can lead to wound dehiscence is a cause of worry.
4. Significant gap (on radiograph) is noted at the proximal aspect of the osteotomy site after de-rotation and it would be nice to know if authors feel that the wedge of bone removed from the lateral side can be put back as a graft on the medial side if significant gap is noted.
5. Figure 4 – The fibular osteotomy demonstrated in the figure 4 is a medial to lateral downward sloping short oblique osteotomy. However, in the antero-posterior radiograph of the index case the fibular osteotomy appear as a transverse cut. Readers would be interested to know about the best direction for the fibular osteotomy which would help in translation of the fibula during de-rotation.
6. Discussion – It would be informative if authors can discuss the merits and demerits of RAB’s osteotomy which seems to be simpler than ‘Z’ osteotomy. Also, post-operative correction can be adjusted if under or over correction is noted by wedging of the cast in RAB’s osteotomy.
7. Conclusion – I/we feel ‘Z’ osteotomy is not simpler than RAB’s osteotomy. The conclusion may be modified describing ‘Z’ osteotomy as another new technique to correct the mechanical axis.
8. It would be worth mentioning that a longer follow up is required to know the status of the proximal tibial medial physis and if the correction of the deformity is maintained till skeletal maturity.

We have read this submission. We believe that we have an appropriate level of expertise to confirm that it is of an acceptable scientific standard.

# Peer review: Referee response for version 1

**DOI:** 10.5256/f1000research.7274.r14311

**Published:** 2016-06-14

**Authors:** Nikolaos Gougoulias

**Affiliations:** 1 Trauma & Orthopaedics Department, Frimley Park Hospital, Frimley, Surrey, UK

This is an interesting, nicely illustrated, article describing a surgical technique that can be applied for the management of Blount's disease. Although most surgeons use external fixation for the correction of such deformities, the current article addresses another possibility, namely acute correction and internal fixation. Some patients (especially small children) might not tolerate external fixation, thus this technique offers another tool in the surgeon's armamnetarium. Therefore it is a contribution to the literature if alternative techniques are presented. The postoperative result is also nicely illustrated.

I have read this submission. I believe that I have an appropriate level of expertise to confirm that it is of an acceptable scientific standard.
